# Tubercular Osteomyelitis of the Orbit Presenting as Periorbital Cellulitis

**DOI:** 10.18502/jovr.v17i1.10181

**Published:** 2022-01-21

**Authors:** Shruti Bhattacharya, Usha K Raina, Shantanu K Gupta, Manisha Mishra, Varun Saini, Brajesh Kumar

**Affiliations:** Department of Ophthalmology, Guru Nanak Eye Centre, Maulana Azad Medical College, New Delhi, India

**Keywords:** Orbital Tuberculosis, Periorbital Cellulitis, Tubercular Osteomyelitis

## Abstract

**Purpose:**

Osteomyelitis of the orbital bones presenting as an orbital cellulitis is a rare form of extrapulmonary tuberculosis (TB). We report a rare case of tubercular osteomyelitis of the orbital bones presenting as a periorbital cellulitis.

**Case Report:**

A seven-year-old female child presented to our tertiary eye care center with swelling involving the right eyelids and the right cheek for two months. She had been provisionally diagnosed elsewhere as pre-septal cellulitis and had been given oral antibiotics. We clinically diagnosed her as orbital cellulitis, but her non-responsiveness to intravenous antibiotics prompted us to get a contrast enhanced computed tomography (CECT) of the orbit and paranasal sinuses, which was suggestive of tubercular etiology. However, the patient had no foci for TB elsewhere. We used a relatively new, but rapid test, called Cartridge-based Nucleic Acid Amplification Test (CBNAAT) on the pus aspirate which was positive for TB. Thereafter, the patient was started on anti-tubercular treatment to which she responded wonderfully.

**Conclusion:**

A high index of suspicion should be kept for TB infection in cases of orbital cellulitis with unusual clinical behavior in an endemic region such as India.

##  INTRODUCTION

Tuberculosis (TB) is a major cause of morbidity and mortality in developing nations. It is a multisystem infectious disease caused by Mycobacterium TB. TB may be pulmonary or extrapulmonary depending on whether lungs are the primary site of infection or not.

Ocular tuberculosis (OTB) is a manifestation of extrapulmonary TB. It is considered rare even in endemic areas. OTB may be primary or secondary. When the eye is the initial portal of entry of the bacteria, it is considered a primary ocular TB. In secondary ocular TB, the involvement of the eye is due to spread from hematogenous route or from adjacent structures.^[1]^ Ocular TB may have myriad presentations depending upon the site of infection as well as the severity. However, uveal involvement is more common because of its high blood supply.^[2,3]^

Orbital TB is more commonly seen in children with a female predisposition. It is insidious in onset and is usually unilateral.^[4]^ It may present as unilateral proptosis/draining sinus tract/orbital swelling/lid swelling/radiographic features of bony destruction.

We present a rare case of tubercular osteomyelitis of the orbital bones masquerading as periorbital cellulitis.

##  CASE REPORT

A seven-year-old female child presented to our tertiary eye care center with tender swelling involving the right eye and the right cheek for two months. This was not associated with decrease in vision, discharge, photophobia, or fever.

On examination, the swelling involved the right upper and lower eyelid along with the right upper cheek; it was firm in consistency, tender and warm to touch, erythematous, with negative fluctuation tests, and no pus points or discharging sinuses (as shown in Figures 1A & 1B). Visual acuity was 6/6 in both eyes. Anterior as well as posterior segment examination of the eye were unremarkable. Despite the extensive edema, extraocular movements were full and free. Direct and consensual light reflexes were normal. A note was made of the enlarged submandibular lymph nodes. These were 2 × 2 cm, non-tender, non-conglomerated, firm in consistency, not fixed to the overlying skin. The overlying skin was not red and non-tender. The child was otherwise afebrile and had no other systemic complaints.

Furthermore, the patient was being treated with oral antibiotics (no records brought) at a peripheral center for the past one month before presentation to our center, with a steady increase in the size of the swelling. History elicited from the attendants showed their noncompliance with the prescribed treatment. Thereafter, the child was admitted for a course of intravenous antibiotics, with the provisional diagnosis being orbital cellulitis. However, poor response to intravenous antibiotics even after one week prompted us to investigate further. A contrast enhanced computerized tomography of the orbit and the paranasal sinuses (PNS) was ordered which showed a peripherally enhancing collection in the anterolateral aspect of the right maxillary sinus and zygomatic arch, extending superiorly along the lateral aspect of right orbit, showing lytic erosive changes, along with periosteal thickening and bony sequestration in the zygoma (axial scan shown in Figure 3A & 3B). These radiographic features were suggestive of TB.

The child had no history of fever, night sweats, no previous history of TB, and no history of contact with TB. In addition, the swelling was not like a typical tubercular cold abscess and the lymph nodes were not matted nor fixed to the skin. But in view of high endemicity of TB in our country, it was still kept on top of our differentials.

Except for a raised erythrocyte sedimentation rate (ESR), the complete blood count and liver function tests were unremarkable. The tuberculin sensitivity test (TST) showed 20 mm after 48 hr - however this is nonspecific. Fine needle aspiration cytology (FNAC) was done from the swollen submandibular lymph nodes – it showed necrotic changes but no granuloma or acid fast bacilli (AFB). Sputum as well as gastric aspirate microscopy for AFB showed no evidence of AFB.

Antigravity aspirate of the swelling was done with a 26-gauge needle, under local anesthesia. Around 1 ml of the watery yellowish aspirate was then sent for gram stain, bacterial and fungal cultures, and a relatively new test – the Cartridge-based Nucleic Acid Amplification Test (CBNAAT), also known as the Genexpert test. CBNAAT turned out to be positive, thus confirming the diagnosis of primary tubercular osteomyelitis of the right maxilla; the bacterial and gram stain reports were negative, thus ruling out secondary superinfection. The child was put on a four-drug anti-tubercular treatment (isoniazid [10 mg/kg] rifampicin [15 mg/kg], ethambutol [20 mg/kg], and pyrazinamide [35 mg/kg]) and responded well to the treatment, by complete shrinkage of the swelling within six weeks [Figures 2A & 2B].

##  DISCUSSION

Tuberculosis (TB) is a major health problem in developing countries. However, even in endemic areas, orbital TB is a rare entity. Orbital TB can be diagnosed using the following criteria: (1) clinical/radiological or histological evidence of TB from the orbital lesion associated with evidence of TB elsewhere, (2) demonstration of AFB in the orbital lesion, (3) isolation of mycobacteria in culture from biopsied tissue, and (4) demonstration of mycobacterial infection by doing a PCR on the biopsied tissue.^[5]^

Orbital TB has been clinically classified into five categories: classical periostitis, orbital tuberculoma with no bony destruction, orbital TB with evidence of bony destruction (not classified as classical periostitis), orbital TB as a result of spread from paranasal sinuses and dacryoadenitis. Patients with classical periostitis present with chronic ulceration or discharging sinus in the periorbital region. There may or may not be evidence of bony erosion or sclerosis radiographically. Orbital tuberculoma presents with palpable mass, proptosis, or diplopia. When radiologic evidence of bony destruction, osteolytic changes, or erosion is seen, it is classified as orbital TB with bony destruction. Maxillary sinus is the most commonly involved sinus from which TB can spread to orbit. Such patients usually present with proptosis and dystopia of the globe. Patients with tubercular dacryoadenitis present with a mass in the lacrimal region and regional lymphadenopathy.^[5,6]^ Osteomyelitis of tubercular origin involving the orbital bones is rare. Mandible is the most common craniofacial bone affected by TB.^[7]^ This condition is usually secondary to a primary tubercular lesion elsewhere, such as the lungs and the abdomen. However, primary involvement of these bones have also been reported.

Our patient presented with swelling involving the right periorbital region extending up to the right cheek. She was given a course of antibiotics to which no response was seen and the patient was investigated for other etiologies including TB. Our differentials at this time were local cellulitis, neoplasia, pseudotumor, easophillic granuloma, and TB, and she was investigated for all of these diagnoses.

Incision and drainage of the swelling was not done because an incision and drainage (I&D) would lead to a persistently discharging sinus in case it was tubercular in etiology.

There was no histopathology evidence of TB but radiologically the lesion was suggestive of TB which was done a week later after no response to intravenous antibiotics. We waited for a week before imaging was done because the history of treatment given to her was very uncompliant and no intravenous antibiotics were given before the patient presented to us.

We did keep in mind other conditions causing lytic changes in the orbit such as neoplastic, inflammatory, and rare causes like eosinophilic granulomas. Our diagnosis of TB was confirmed by CBNAAT test done on the sample from the orbital lesion. Thereafter, the patient was started on anti-tubercular drugs.

We should keep a high index of suspicion for orbital TB in cases like ours especially in endemic areas such as India. The patient should be investigated for the same. However, the diagnosis is often challenging in cases of extrapulmonary TB as the number of TB bacilli is often low in these sites.^[8]^ Although the demonstration of AFB in culture is considered the gold standard for diagnosis, it may take up to six to eight weeks, thus delaying the treatment.^[9]^

Molecular diagnostics such as nucleic acid amplification test play an important role in cases of extrapulmonary TB. A new breakthrough has been the CBNAAT, also known as the Genexpert test. This test not only detects Mycobacterium DNA but also reveals whether it is sensitive to rifampicin or not, thereby identifying multidrug-resistant TB cases. The main advantage of CBNAAT is the speed and accuracy with which it works. It has been shown to have comparable results with culture (which may take up to six to eight weeks). However, it has its own set of flaws such as high cost, need for a stable electricity supply, replenishment of the cartridges every 18 months, and a stable temperature ceiling.^[10]^

Interferon gamma release assays can also be used as an aid to diagnosis. These tests are based on the production of interferon gamma by T cells specific to antigens of tubercular bacilli. However, they are less sensitive for detection of latent TB infection.^[1]^

Radiological features of orbital TB include destruction of bone, most commonly the frontal and sphenoidal bones. There may or may not be associated bony sclerosis, inflammation, abscess formation which may extend to infratemporal fossa. Lacrimal gland involvement may also be seen. Involvement of lateral wall is usually suggestive of hematogenous route of infection while involvement of medial wall is indicative of spread from adjoining paranasal sinuses. There may be associated pre-septal thickening.^[11]^

Treatment of tubercular osteomyelitis is mainly done using anti-tubercular drugs. Surgery is indicated in cases with extensive destruction, presence of secondary infection, and intracranial involvement. Our patient was managed medically by anti-tubercular treatment.

To conclude, although orbital TB is rare, it should still be kept in mind while seeing patients with orbital lesions in an endemic country, and new tests such as CBNAAT should be used more frequently because of increased sensitivity and rapid diagnosis.

##  Financial Support and Sponsorship

Nil.

##  Conflicts of Interest

There are no conflicts of interest.
